# Investigation of fingolimod-induced lymphocyte sequestration on inflammatory response and neurological damages after cardiac arrest

**DOI:** 10.1186/s40635-024-00645-4

**Published:** 2024-07-01

**Authors:** Yara Abi Zeid Daou, Fanny Lidouren, Antoine Bois, Naoto Watanabe, Ali Jendoubi, Estelle Faucher, Mathieu Surenaud, Sophie Chateau-Joubert, Sophie Hue, Bijan Ghaleh, Matthias Kohlhauer, Renaud Tissier

**Affiliations:** 1 Univ Paris Est Créteil, INSERM, IMRB, 94010 Créteil, France; 2 Ecole Nationale Vétérinaire d’Alfort, IMRB, AfterROSC Network, 7 avenue du Général de Gaulle, 94700 Maisons-Alfort, France; 3 Service de Médecine Intensive-Réanimation, Hôpitaux Universitaires Paris Centre, Hopital Cochin, Paris, France; 4 Vaccine Research Institute, Univ Paris Est-Creteil, 94000 Creteil, France

**Keywords:** Cardiac arrest, Resuscitation, Lymphocyte, Sepsis-like, Inflammation, Neurological dysfunction

## Abstract

**Background:**

A sepsis-like syndrome is known to occur after cardiac arrest, leading to cerebral infiltration by white blood cells (WBC). We hypothesized that pharmacological sequestration of WBC, and more specifically lymphocytes within lymphoid tissues, could reduce the cerebral infiltration by these inflammatory cells and subsequent acute brain injury in a porcine model of cardiac arrest. Lymphocyte sequestration was induced by the sphingosine-1 phosphate receptors agonist fingolimod.

**Methods:**

In a first set of experiments, anesthetized pigs underwent a sham instrumentation with no cardiac arrest (*n* = 4). They received an administration of fingolimod (1 mg/kg, i.v.) in order to confirm its effect on WBC. In a second set of experiments, animals randomly received fingolimod or saline two hours prior to an episode of ventricular fibrillation (14 min) with subsequent resuscitation (*n* = 6 in each group). Neurological injury was assessed 24 h after resuscitation.

**Results:**

In the first set of experiments, WBC and blood lymphocyte counts were significantly reduced by − 61 ± 10% and − 75 ± 6% two hours after fingolimod administration. In the second set of experiments, blood lymphocyte counts, but not WBC, were also significantly reduced after cardiac arrest in Fingolimod vs Control group. However, most cytokine blood levels were not different among groups, including Interleukin (IL)-1ra, IL-8 or IL-18 blood levels. A difference was only observed for IL-6, which decreased in Fingolimod vs Control (e.g., 5.6 ± 4.8 vs 59.4 ± 20.6 pg/ml at 2 h after cardiac arrest, respectively; p = 0.126). Neurofilament light chain (NFL) blood levels were not different among groups (57 ± 25 vs 84 ± 41 pg/ml in Fingolimod vs Control at 6 h after resuscitation, respectively). After awakening, 3 and 2 animals were prematurely euthanized for ethical reasons due to recurrent seizures in Fingolimod and Control groups, respectively. At Day 1, neurological dysfunction score was not different between groups (87 ± 7 vs 87 ± 5% in Fingolimod vs Control, respectively). Conversely, a decrease in the number of CD3 + cells was observed in the brain of surviving animals in Fingolimod vs Control group (3.10 ± 0.50 vs 7.53 ± 0.57 CD3 + cells/field, respectively; *p* = 0.0286).

**Conclusion:**

Fingolimod-induced WBC sequestration, and more specifically lymphocytes sequestration, did not improve clinical neurological dysfunction following cardiac arrest although it reduced cerebral infiltration by lymphocytes.

**Supplementary Information:**

The online version contains supplementary material available at 10.1186/s40635-024-00645-4.

## Background

Despite the significant progress that has been made in the treatment of out-of-hospital cardiac arrest, surviving patients experience a multitude of complications related to whole-body ischemia–reperfusion injury and “sepsis-like syndrome” [1–3]. Immunomodulating therapies, such as interleukin (IL)-6 receptor antibodies, have been evaluated in patients but failed to improve the neurological outcome until now [4, 5]. Conversely, preclinical studies have demonstrated that the cerebral infiltration by circulating white blood cells (WBC), and more specifically lymphocytes, occurs very early after return of spontaneous circulation (ROSC) [6, 7].

In the present study, we aimed to determine whether the pharmacological sequestration of WBC within lymphoid tissues could prevent brain injury after cardiac arrest. To achieve this goal, we used fingolimod, a sphingosine 1 receptor modulator. This drug is the first oral treatment approved for a medical use in relapsing forms of multiple sclerosis. It is metabolized by sphingosine kinase into fingolimod-phosphate (fingolimod-P) [8], which binds to lymphocytes’ sphingosine 1 phosphate receptors (r-S1P). This blocks the ability of lymphocytes to egress from lymph nodes resulting in their sequestration within lymphoid tissues, subsequently reducing the total number of circulating lymphocytes in peripheral blood [9, 10]. After regional cerebral ischemia, fingolimod has been shown to provide a potent neuroprotective effect in rodents [11]. After experimental cardiac arrest, fingolimod was also shown to activate survival pathways and attenuate myocardial inflammation, oxidative stress and apoptosis in rats [12]. However, the impact of fingolimod and more broadly, the effect of pharmacologically induced sequestration of WBC, on cerebral brain injury has not been investigated after cardiac arrest to our knowledge. It could also support the repurposing of fingolimod for post-cardiac arrest treatment.

Accordingly, the primary objective of the present study was to determine whether fingolimod can mitigate the inflammatory response and offer neuroprotection following cardiac arrest in pigs, considering its ability to reduce circulating lymphocytes counts. In a first set of experiments, we observed that fingolimod reduced WBC and lymphocyte counts in Sham conditions, i.e., without inducing cardiac arrest, within two hours after its administration. Then, in a second set of experiments, we evaluated the neuroprotective effect of fingolimod when administered two hours before cardiac arrest, in order to achieve lymphocyte sequestration before reperfusion and therefore mechanistically evaluate the link between early lymphocyte infiltration and acute brain injury.

## Methods

All experiments were approved by the local ethical committee for animal research (Comité d’Ethique n°16 Anses – ENVA – UPEC; APAFIS #32855-2021090315125530 v2) and comply with French and European regulations concerning the use of laboratory animals.

### Animal preparation

Female swine (Lebeau, Gambais France) were anesthetized using a combination of tiletamine and zolazepam (both 10 mg/kg i.m.) followed by propofol (10 mg/kg/h, i.v.). Methadone was administered for analgesia (0.75 mg/kg, i.m.). After endotracheal intubation, animals were submitted to conventional mechanical ventilation (FiO2 = 30%, tidal volume = 8 mL/kg, respiratory rate = 20 cycles/min; Monnal T60®, Air Liquide, Antony, France).

Using the Seldinger technique, two central catheters were inserted into the femoral vessels for the monitoring of arterial and right atrial blood pressure with a dedicated pressure transducer (Millar®, SPR-524, Houston, TX, USA). Two additional central catheters (arterial and venous) were inserted for the continuous evaluation of cardiac output (CO) by thermodilution (PiCCO2*®,* PC8500, Germany). The device was calibrated prior to and 1, 2 and 6 h after cardiac arrest by 10 ml of cold saline. Fluid loss was compensated with a standardized administration of Ringer lactate (4 mL/kg). Body temperature was maintained at 38.0 ± 0.5°C with a heating pad.

### Experimental groups

**Fig. 1:**
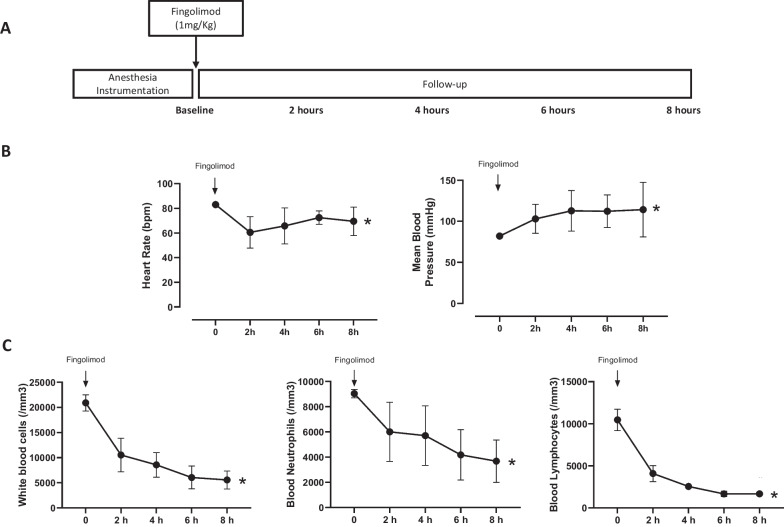
Schematic representation of the first set of experiments with no cardiac arrest (**A** Sham protocol), heart rate, mean arterial pressure (**B**) and white blood cells count (**C**) after fingolimod administration. *p < 0.05 for the time effect of the one-way analysis of variance for repeated measures. Exact p values and post hoc comparisons at each time-point are shown in Supplemental Table 2

During a first set of experiments (Fig. 1A), four swine underwent a Sham procedure, i.e., instrumentation without inducing cardiac arrest. They all received fingolimod (1 mg/kg, i.v.) and were monitored over 8 h to evaluate its impact on blood cell counts and hemodynamics.

**Fig. 2:**
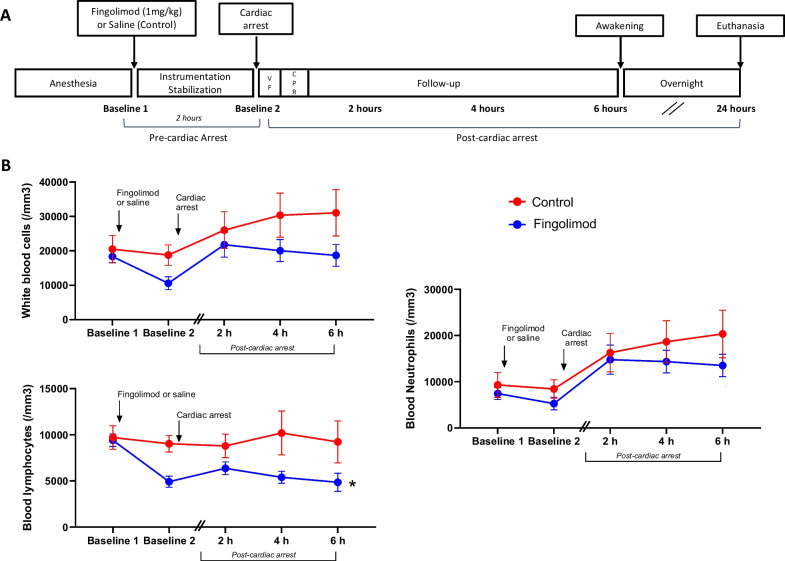
Schematic representation of the second set of experiment in animals submitted to cardiac arrest (**A**) and white blood cells count in this experiment (**B**). *VF* ventricular fibrillation, *CPR* cardiopulmonary resuscitation; *p < 0.05 vs Control group; *n* = 6 in both groups. *p < 0.05 for the group or group x time effect of the two-way analysis of variance for repeated measures. Exact *p* values and post hoc comparisons at each time-point are shown in Supplemental Table 3

During the second of experiments (Fig. 2A), pigs were anesthetized and randomly received fingolimod (1 mg/kg, i.v.) or saline (*n* = 6 in each group). They were instrumented and submitted to cardiac arrest two hours after drug administration. Cardiac arrest was induced by ventricular fibrillation via the delivery of an alternating current (10 V) with a pacemaker probe introduced into the right ventricle and the interruption of mechanical ventilation. After 14 min of untreated ventricular fibrillation, cardiopulmonary resuscitation (CPR) was initiated with continuous external chest compressions using an automated device (100 compressions/min; Thumper; Michigan Instruments, Grand Rapids, MI) and resumption of mechanical ventilation (FiO2 = 100%; respiratory rate = 10 cycles/min). After 2 min of chest compression, epinephrine (10 µg/kg i.v.) was administered and external defibrillation were attempted every 3 min until ROSC. Norepinephrine was administered to maintain mean arterial blood pressure above 75 ± 5 mmHg during follow-up if needed. Animals received a second administration of methadone 6 h after the first injection (0.75mg/kg, i.m.). After 6 h of mechanical ventilation, animals were weaned from artificial ventilation and awaken. A fentanyl patch (100 µg/h) was applied on their skin to maintain analgesia overnight. In case of recurrent seizures after awakening, animals were euthanized for ethical reasons before being returned to their cages. Other animals were housed in their cages overnight before undergoing a neurological evaluation 24 h after cardiac arrest. At the end of the protocol, additional blood samples were collected and animals were examined before euthanasia with a pentobarbital overdose (60 mg/kg i.v.). Brains were withdrawn and fixed with 5% paraformaldehyde for further histological analyses.

### Investigated parameters

Mean arterial blood pressure, heart rate and cardiac output were continuously monitored until awakening and weaning from mechanical ventilation. Arterial blood samples were collected at various time points to assess blood gases and blood cell counts. Additional blood samples were collected to obtain serum samples that were stored at -80°C to further evaluate cytokine and neurofilament light chain (NFL) blood concentrations. Briefly, cytokine concentrations were assessed using a multiplex evaluating tumor necrosis factor-α (TNF-α), interleukin (IL)-1ra, IL-6, IL-8, IL-18, matrix metalloproteinase-1 (MMP1) and CD31 levels (8-plex R&D systems # LXSAPM-08, L151732; Bio-Plex 200, Bio-Rad). NFL concentrations were also evaluated by a dedicated porcine kit (Neuro 2-Plex B Advantage Kit #103520; Simoa SR-X, Quanterix).

Neurological dysfunction was blindly evaluated 24 h after cardiac arrest using a clinical score (Supplemental Table 1). At the end of the protocol, Fluorojade C staining was used to identify degenerating neurons on the harvested brains in 6 non-overlapping fields within the cortex of animals surviving until Day 1 after cardiac arrest (Merck Millipore, Burlington, MA). CD3 + cells were also stained and counted using a monoclonal antibody against a specific cell surface antigen (Anti-CD3 #A0452, Dako). We focused on CD3 + cells since T cells, expressing this surface antigen, were shown to be critical for brain injury after cardiac arrest [6, 7].

### Statistical analysis

Data are presented as mean ± standard error of the mean (SEM). In the first set of experiment, continuous variables were compared to baseline using a one-way analysis of variance for repeated measures (ANOVA). Exact p values are displayed in Supplemental Table 2. In the first second of experiment, continuous variables were compared between groups using a two-way ANOVA, followed by a Fisher’s LSD test. Post hoc comparisons were made between groups at each time-point, but not between timing within group. Exact p values are displayed in Supplemental Table 3. Neurological dysfunction, number of degenerating neurons and CD3 + cells count in the brain were compared between groups using a non-parametric Mann–Whitney test. Statistical significance was set as p < 0.05. GraphPad Prism® software (GraphPad® Software, La Jolla, CA, USA) was used for all statistical analyses.

## Results

### Investigation of the acute effect of fingolimod in Sham conditions

In the first set of experiment, four pigs (33 ± 2 kg) were submitted to sham instrumentation without cardiac arrest induction. As illustrated in Fig. 1B, heart rate decreased after fingolimod administration (e.g., 61 ± 7 vs 81 ± 1 beats/min at 2 h after fingolimod vs baseline, respectively). This was associated with an increase in mean arterial pressure (e.g., 103 ± 10 vs 82 ± 2 mmHg at 2 h after fingolimod vs baseline, respectively).

As illustrated in Fig. 1C, WBC and blood lymphocyte counts significantly decreased after fingolimod administration (- 61 ± 10 and—75 ± 6% at 2 h and − 73 ± 10 and − 84 ± 2% at 6 h after its administration vs baseline, respectively). A reduction in neutrophil count was also observed but achieved a milder magnitude (− 39 ± 27% and − 56 ± 23% at 2 and 6 h after fingolimod vs baseline, respectively).

### Cardiopulmonary resuscitation parameters in animal submitted to cardiac arrest

In the second set of experiments, 18 pigs were submitted to cardiac arrest (n = 9 in both Fingolimod and Control group). Body weight (33 ± 1 vs 34 ± 3 kg) and body core temperature at baseline (37.8 ± 0.2 vs 37.7 ± 0.2 °C) did not differ between Fingolimod and Control groups, respectively. During CPR, the cumulative dose of epinephrine and the number of external defibrillation shocks was not different between Fingolimod and Control groups (1.18 ± 0.46 vs 1.21 ± 0.25 mg and 5.6 ± 2.3 vs 6.8 ± 1.7 shocks, respectively). ROSC was obtained in 6 animals in each group, which were finally included in the subsequent analysis for post-cardiac arrest alterations.

### Fingolimod reduces WBC count after cardiac arrest

As illustrated in Fig. 2B, WBC, blood lymphocyte and neutrophil counts were similar in both groups at the first baseline, i.e., before cardiac arrest and investigational drug administration. Lymphocyte counts significantly decreased in Fingolimod vs Control group at the second baseline (two hours after fingolimod) and after cardiac arrest. Conversely, WBC and neutrophil counts were not significantly different in Fingolimod vs Control.

### Fingolimod does neither improve hemodynamic nor gas exchange after cardiac arrest

**Fig. 3:**
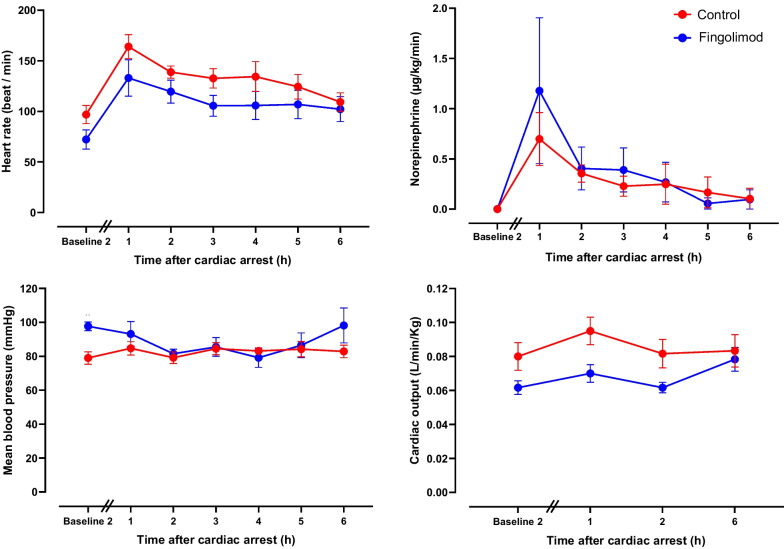
Hemodynamic parameters and amount of administered norepinephrine in animals submitted to cardiac arrest after the administration of fingolimod or vehicle Baseline 2 represents measurement 2 h after investigational drug administration; values prior to drug administration are not available since central catheters were not yet inserted at the time of administration; *n* = 6 in both groups. Exact *p* values of the two-way analysis of variance for repeated measures are shown in Supplemental Table 3

As illustrated in Fig. 3, mean arterial pressure was higher in Fingolimod vs Control group at the second baseline (98 ± 3 vs 79 ± 4 mmHg two hours after investigational drug administration, respectively), but this was not significantly different (p value for group x time interaction = 0.0835). Hemodynamic parameters at the first baseline were not recorded since drugs were administered before central catheter insertion, but this is consistent with the hemodynamic effect observed with fingolimod in Sham conditions in the first set of experiment. Heart rate also decreased in Fingolimod vs Control, but it did not achieve statistical significance (p value for group effect = 0.102). This is in line with a trend toward lower cardiac output since the second baseline. After cardiac arrest, hemodynamic parameters were not significantly different between groups, as well as the administered doses of norepinephrine (e.g., 0.40 ± 0.23 vs 0.36 ± 0.09 µg/kg/min at 2 h following cardiac arrest in the Fingolimod and Control group, respectively).

As shown in Table 1, blood pH, gases, bicarbonate and lactate levels were not different among groups. A metabolic acidosis was observed in both groups with increased blood lactate levels and decreased bicarbonates and pH after cardiac arrest (e.g., 7.17 ± 0.6 vs 7.02 ± 0.04 of pH at 30 min after cardiac arrest in the Fingolimod and Control group, respectively).

**Table 1:** Blood gases, pH, lactate and bicarbonate levels

	Baseline	Time after ROSC					
		30 min	60 min	120 min	180 min	240 min	360 min
*Arterial blood pH*							
Control	7.43 ± 0.02	7.02 ± 0.04	7.14 ± 0.04	7.27 ± 0.03	7.31 ± 0.04	7.33 ± 0.05	7.31 ± 0.04
Fingolimod	7.43 ± 0.02	7.17 ± 0.06	7.23 ± 0.07	7.31 ± 0.05	7.35 ± 0.06	7.36 ± 0.05	7.35 ± 0.02
*Arterial blood PaCO*_*2*_* (mmHg)*							
Control	42 ± 2	60 ± 1	56 ± 2	47 ± 3	49 ± 3	47 ± 4	51 ± 3
Fingolimod	40 ± 0.8	47 ± 2.88	46 ± 3	41 ± 4	41 ± 3	43 ± 2	42 ± 6
*Arterial blood PaO*_*2*_* (mmHg)*							
Control	163 ± 9	110 ± 3	118 ± 5	138 ± 13	134 ± 8	130 ± 7	133 ± 6
Fingolimod	177 ± 6	164 ± 41	138 ± 10	145 ± 9	137 ± 14	148 ± 19	1252 ± 12
*Bicarbonate levels (mmol/L)*							
Control	27 ± 1	16 ± 1	18 ± 2	21 ± 2	24 ± 1	24 ± 1	25 ± 1
Fingolimod	26 ± 1	17 ± 2	19 ± 2	21 ± 2	23 ± 2	24 ± 2	22 ± 3
*Lactate levels (mmol/L)*							
Control	2.7 ± 0.3	14.4 ± 1.1	12.1 ± 1.1	7.3 ± 0.9	5.2 ± 1.6	4.4 ± 2.1	3.9 ± 1.3
Fingolimod	3.6 ± 0.6	12.4 ± 1.7	10.2 ± 2.1	7.7 ± 2.5	6.2 ± 2.3	5.0 ± 2.0	5.0 ± 2.3

*PaCO*_*2*_ partial pressure of carbon dioxide,* PaO*_*2*_ partial pressure of oxygen

### Fingolimod only transiently reduces IL-6 blood levels among a wide panel of cytokine

**Fig. 4:**
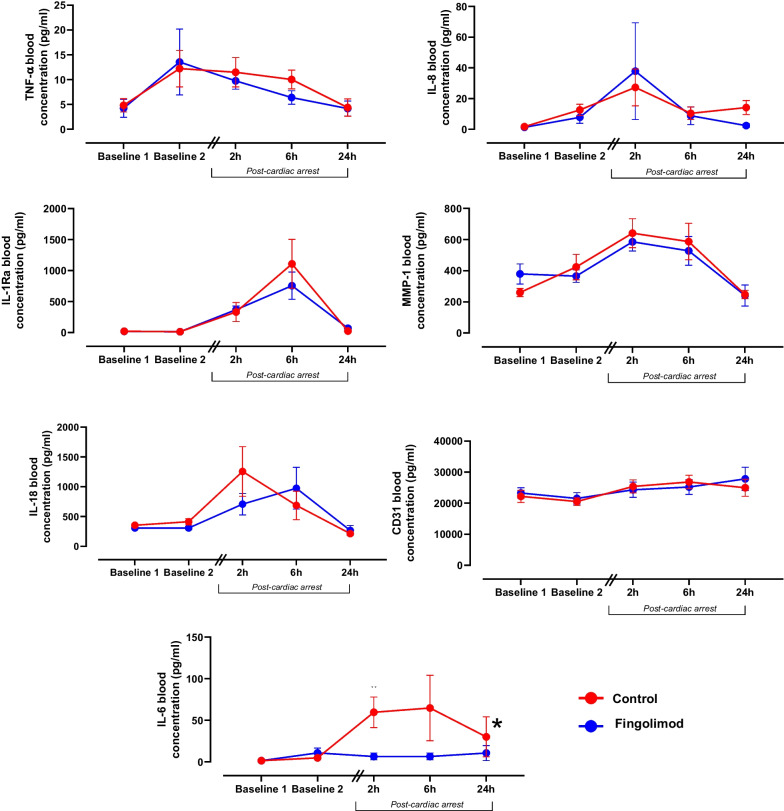
Blood cytokine concentrations in animals submitted to cardiac arrest after the administration of fingolimod or vehicle. TNF-α, tumor necrosis factor-α; IL-1ra, interleukin-1ra; IL-6, interleukin-6; IL-8, interleukin-8; IL-18, interleukin-18; MMP-1, matrix metalloproteinase-1; *p < 0.05 vs Control group; *n* = 6 in both groups. *p < 0.05 for the group or group x time effect of the two-way analysis of variance for repeated measures. Exact p values and post hoc comparisons at each time-point are shown in Supplemental Table 3

As illustrated in Fig. 4, blood levels of TNF-α, IL-1ra, IL-6, IL-8, IL-18, MMP1 and CD31 were not different among groups before (baseline 1) or two hours after the administration of fingolimod or saline (baseline 2). After cardiac arrest, blood concentrations of most of the investigated cytokines increased in the Control group, except for CD31 and TNF-α. The pattern of alteration was similar in the Fingolimod group, except for IL-6 blood levels that were significantly decreased vs Control at 2 and 6 h following cardiac arrest (e.g., 5.6 ± 4.8 vs 59.4 ± 20.6 pg/ml at 2 h following cardiac arrest in Fingolimod vs Control, respectively).

### Fingolimod does neither improve survival, neurological dysfunction nor NFL blood levels

**Fig. 5:**
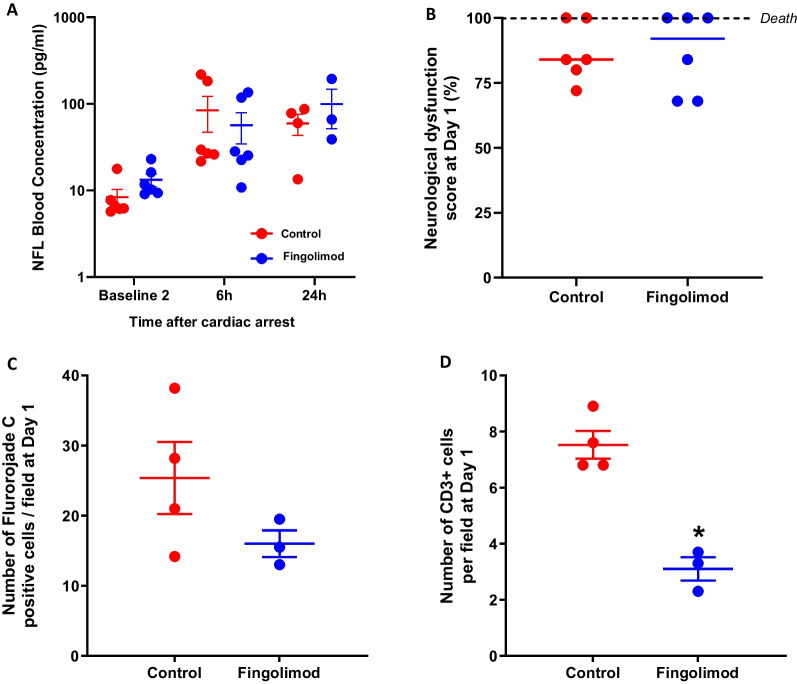
Neurofilament light chain blood levels (**A**), neurological dysfunction score at Day 1 (**B**; 0 and 100% meaning normal neurological condition and death, respectively), number of Fluorojade C-positive neurons per histological field in the cortex of surviving animals at 24 h after cardiac arrest (**C**) and number of CD3 + cell per histological field in the cortex in the same animals (**D**). *p = 0.0286 vs Control

As illustrated in Fig. 5A, NFL blood levels similarly increased after cardiac arrest in both Fingolimod and Control groups (e.g., 57 ± 25 vs 84 ± 41 pg/mL at 6 h following cardiac arrest in Fingolimod vs Control, respectively).

After weaning from mechanical ventilation and awakening, 3 and 2 animals were euthanized for recurrent seizures in the Fingolimod and Control groups, respectively. As illustrated in Fig. 5B, neurological dysfunction was not significantly different among groups at Day 1.

### *Fingolimod reduced the number of CD3* + *cells infiltrating the brain in survivors*

As illustrated in Fig. 5C, the total number of degenerating neurons was attenuated in the 3 and 4 animals surviving until Day 1 in Fingolimod vs Control but no statistical significance was observed. Interestingly, the number of CD3 + cells significantly decreased in Fingolimod vs Control in those same animals (Fig. 5D), respectively.

## Discussion

In the present study, fingolimod potently reduced blood lymphocytes counts after cardiac arrest but did not improve survival and clinical neurological dysfunction. A trend toward reduced brain injury was observed regarding the histological number of degenerating neurons and a significant decrease in brain CD3 + cells. Our findings suggest that blood lymphocyte sequestration in lymphoid tissue reduces the cerebral infiltration by lymphocytes, but is not sufficient to provide neuroprotection regarding clinical outcome.

Our first finding confirms the impact of fingolimod on lymphocyte counts. In “non-cardiac arrest animals” (first set of experiments), the effect was observed within 2 h after fingolimod administration and achieved a ceiling after 6 h. Fingolimod had a major impact in attenuating blood lymphocytes but WBC and neutrophil counts were also reduced. This is consistent with previous findings in patients that demonstrated a maximal blood lymphocyte count reduction after 8 h following i.v. administration [13]. Blood neutrophil counts were not investigated in the latter study, neither were other WBC populations. In “animals submitted to cardiac arrest” (second set of experiments), fingolimod only significantly reduced blood lymphocyte count, which was the most important parameter since this population is supposed to be a major component in brain injury after cardiac arrest [6, 7]. This reduction was observed prior to cardiac arrest, which allowed to evaluate the impact of blood lymphocyte count reduction since the onset of CPR.

Interestingly fingolimod did not significantly reduce the circulating levels of most pro- or anti-inflammatory cytokines in the present study, despite the reduction in WBC counts as compared to Control. A significant reduction was only observed for IL-6 blood levels, as well a trend toward a delayed increase in IL-18 levels with fingolimod, both suggesting decreased inflammatory response. Circulating levels of cytokines do not only depend upon blood cell counts, which could explain our results. In patients, fingolimod was indeed not associated with significant changes in IL-6, IL-8 and TNF-α blood levels during a long-term treatment from relapsing forms of multiple sclerosis [14]. It would be more relevant to evaluate the tissular concentration of cytokines but this was not feasible in the present study, due to the difficulty to obtain multiple brain samples in the same animals very early after cardiac arrest.

More importantly, the reduction in blood lymphocyte counts with fingolimod led to a reduction in cerebral infiltration by CD3 + cells in animals surviving during 24 h. This demonstrated a link between circulating lymphocytes and, at least, brain T cells infiltration. This is also in line with reduced IL-6 blood levels and delayed increase in IL-18 levels with fingolimod. One would argue that WBC other than T cells could probably contribute to neuroinflammation, but the latter cells were shown to be of a particular importance in brain injury following cardiac arrest [6], stroke [15] or intracerebral hemorrhage [16]. For instance, Deng et al. observed a rapid lymphocyte infiltration by pro-inflammatory Th40 cells, that are strongly associated with autoimmunity, into the brain parenchyma after cardiac arrest in mice (CPR) [6]. In a swine model of cardiac arrest, cerebral infiltration by cytotoxic lymphocytes was also observed rapidly following asphyxial cardiac arrest [7]. Similar results were observed in rabbits after cardiac arrest [17], suggesting a species-independent mechanism.

Interestingly, the effect of fingolimod on blood lymphocytes and brain CD3 + cells was not associated with improved neurological outcome after cardiac arrest. This is not consistent with previous results in stroke models, which demonstrated a significant improvement in neurological outcomes [11]. Neuroprotective effects were also observed in an experimental model of rodent intracerebral hemorrhage [16]. One could argue that this is related to an insufficiently powered study, since we have a trend toward reduced number of degenerating fluorojade C neurons in the brain of surviving animals. However, we did not observe any impact regarding NFL blood levels, which allow us to assess brain injury, even in prematurely euthanized animals presenting severe and potentially severe hypoxic–ischemic encephalopathy. A discrepancy between clinical and histological outcome could explain our findings, as observed in other animal studies after cardiac arrest [18]. One would also argue that the lack of benefit of fingolimod on neurological outcome could be attributed to the severity of the acute brain injury in our study. We indeed used a prolonged period of no-flow (14 min), based on preliminary experiments testing different durations and aiming at obtaining a reproducible and severe injury in Control conditions (unpublished data). However, it is reasonable to speculate that fingolimod provided either no or little benefits on the neurological outcome. This suggests that a “selective” WBC reduction, and more specifically a reduction in circulating lymphocytes, is not sufficient to provide strong benefits and should at least be combined with other action mechanism to lead to potential protection after severe post-cardiac arrest brain injury. In any case, our findings does not specifically support the clinical evaluation of fingolimod after cardiac arrest. It is also important to acknowledge that it was administered before cardiac arrest in our study, which is obviously not the therapeutic way of administration.

### Study limitations

Our study has several limitations. First, we have chosen to administer fingolimod before cardiac arrest, for a mechanistic purpose. Accordingly, results could theoretically not be extrapolated to potential findings with a post-cardiac arrest administration, which would be more clinically relevant. Second, we observed mild but significant hemodynamic effects with fingolimod on mean arterial pressure prior to cardiac arrest. Such hemodynamic effects were previously reported [19]. Third, we performed brain histology for the evaluation of cerebral infiltration by T cells. Fluorescence-activated cell sorting (FACS) analyses would have been more accurate for quantification [17]. Fourth, we only tested one dose and one scheme of administration of fingolimod. We cannot rule out that a greater effect on WBC counts and lymphocyte would have induced more pronounced neurological effects. Fifth, our hypothesis was related to the immunomodulatory effect of fingolimod on WBC. However, fingolimod and its phosphorylated form, P-fingolimod, might exert pleiotropic effects on S1P receptors, which are also expressed on oligodendrocytes, astrocytes, neurons, and microglia, as well as vascular endothelial cells. For instance, preclinical studies demonstrated that fingolimod attenuated demyelination via direct effects in the central nervous system during multiple sclerosis [20]. Finally, we only evaluated a limited number of animals in the present study. Findings are hard to extrapolate to a wilder population, including animals with comorbidities or other clinical status. Nevertheless, all these limitations are probably not critical since we finally did not observe a potent effect of fingolimod on neurological outcomes.

## Conclusion

Fingolimod-induced WBC sequestration, and more specifically lymphocytes sequestration, did not improve neurological dysfunction following cardiac arrest. However, it reduced cerebral infiltration by lymphocytes as compared to Control.

### Supplementary Information

Supplementary Material 1.

## Data Availability

Data will be available upon request to corresponding author.
