# Supplementary material for: Investigation of fingolimod-induced lymphocyte sequestration on inflammatory response and neurological damages after cardiac arrest
Source: Intensive Care Med Exp. 2024 Jul 1;12:57. doi: 10.1186/s40635-024-00645-4 (PMC11219599; doi:10.1186/s40635-024-00645-4)
Supplement: Supplementary file 1 — Supplementary Material 1. [file 40635_2024_645_MOESM1_ESM.docx]

**Supplemental Table 1:** Neurological dysfunction grading scale for swine (0%, animal is in a good neurological condition; 100%, death).

| **Signs Description** | | | |
| --- | --- | --- | --- |
| **Appetite** | Total food consumed | 0 |  |
|  | More than 50% of the food consumed | 1 |  |
|  | 50% of the food consumed | 2 |  |
|  | Less than 50% of the food consumed | 3 |  |
|  | No food eaten | 4 |  |
| **Standing position** | Normal standing position | 0 |  |
|  | Unstable when standing | 1 |  |
|  | Standing position with support (leans against the wall) | 2 |  |
|  | Rises in response to stimulation | 3 |  |
|  | Lying down, not responding to stimulation | 4 |  |
|  | Motionless | 5 |  |
| **Head position** | Head up | 0 |  |
|  | Head raised in response to stimulation | 1 |  |
|  | Unable to lift head | 2 |  |
| **Vocalization** | Normal and spontaneous vocalization | 0 |  |
|  | Vocalization in response to stimulation | 1 |  |
|  | No vocalization | 2 |  |
| **Movement** | Walk with vitality | 0 |  |
|  | Spontaneously unstable walking | 1 |  |
|  | Unsteady walking in response to stimulation | 2 |  |
|  | Unable to walk | 3 |  |
| **Motor function of forelimbs** | No motor problems | 0 |  |
|  | Slight weakness (instability during movement) | 1 |  |
|  | Moderate paresis (instability when standing) | 2 |  |
|  | Severe paresis | 3 |  |
|  | Complete paralysis | 4 |  |
| **Motor function of hind limbs** | No motor problems | 0 |  |
|  | Slight weakness (instability during movement) | 1 |  |
|  | Moderate paresis (instability when standing) | 2 |  |
|  | Severe paresis | 3 |  |
|  | Complete paralysis | 4 |  |
| **Face** | Symmetrical Face (during rest and movement) | 0 |  |
|  | Facial paralysis | 1 |  |
| **Total** | **Maximum score** | **25** |  |
|  | **Total over 100%** | **100** |  |

**Supplemental Table 2:** P values of the time effects of the one-way analysis of variance for (ANOVA) repeated measured of the different parameters before and after fingolimod administration in the first set of experiments. When a time effect was observed, post-hoc p values were calculated at each time point fingolimod administration vs baseline.

*In red, p value < 0.05.*

|  | **P values of the one-way ANOVA for repeated measures** | **Post-hoc P values vs baseline**  (Time after fingolimod administration) | | | |
| --- | --- | --- | --- | --- | --- |
|  | Time effect | 2 h | 4 h | 6 h | 24 h |
| White blood cell count | **<0.0001** | **0.0110** | **0.0009** | **0.0003** | **0.0002** |
| Blood lymphocyte count | **<0.0001** | **0.0008** | **0.0079** | **0.0033** | **0.0046** |
| Blod neutrophil count | **0.0076** | 0.2330 | 0.2019 | 0.0619 | 0.4072 |
| Heart rate | **0.0230** | **0.0430** | 0.0633 | 0.0960 | 0.1093 |
| Mean blood pressure | 0.1894 | - | - | - | - |

**Supplemental Table 3:** P values of the group, time and group x time effects of the two-way analyses of variance (ANOVA) for the different parameters in the second set of experiments. When a group or group x time interaction was observed, post-hoc p values of group effect were calculated using a Fisher's PLSD test.

*In red, p value < 0.05.*

|  | **P values of the ANOVA** | | | **Post-hoc P values for group effect in case of significant group or groupe x time effect** | | | | | |
| --- | --- | --- | --- | --- | --- | --- | --- | --- | --- |
|  | Group x time interaction | Time effect | Group effect | Baseline 1 | Baseline 2 | 2h | 4h | 6h | 24h |
| White blood cell count | 0.118 | **0.0058** | 0.1941 | - | - | - |  | - |  |
| Blood lymphocyte count | 0.1947 | 0.1090 | **0.0372** | 0.8734 | **0.0327** | 0.2014 | **0.0134** | **0.0236** |  |
| Blod neutrophil count | 0.4321 | **<0.001** | 0.4017 | - | - | - | - | - |  |
| Heart rate | 0.8489 | **0.0001** | 0.1020 | - | - | - | - | - |  |
| Norepinephrine dose | 0.6137 | **0.0337** | 0.8514 | - | - | - | - | - |  |
| Mean blood pressure | 0.0835 | 0.1781 | 0.2324 | - | - | - | - | - |  |
| Cardiac output | 0.1547 | **0.0321** | 0.0673 | - | - | - | - | - |  |
| TNF-a blood level | 0.6677 | 0.0961 | 0.9025 | - | - | - |  | - | - |
| IL-18 blood level | 0.6902 | **0.0271** | 0.2674 | - | - | - |  | - | - |
| IL-R1a blood level | 0.6389 | **0.0041** | 0.7399 | - | - | - |  | - | - |
| MMP-1 blood level | 0.8995 | **<0.001** | 0.4189 | - | - | - |  | - | - |
| CD31 blood level | 0.7362 | **0.0488** | 0.4527 | - | - | - |  | - | - |
| IL-6 blood level | **0.0480** | 0.1304 | 0.0699 | >0.9999 | 0.7845 | **0.0126** |  | **0.0068** | 0.4238 |
| NFL blood level | 0.9308 | **0.0159** | 0.4941 | - | - |  |  | - | - |
